# Adherence to TB treatment remains low during continuation phase among adult patients in Northwest Ethiopia

**DOI:** 10.1186/s12879-021-06428-6

**Published:** 2021-07-31

**Authors:** Kassahun Dessie Gashu, Kassahun Alemu Gelaye, Binyam Tilahun

**Affiliations:** 1 Department of Health Informatics, Institute of Public Health, College of Medicine and Health Sciences, University of Gondar, Gondar, Ethiopia; 2 Department of Epidemiology and Biostatistics, Institute of Public Health, College of Medicine and Health Sciences, University of Gondar, Gondar, Ethiopia

**Keywords:** Adherence, Continuation phase, Tuberculosis, Ethiopia

## Abstract

**Background:**

Patients’ failure to adhere to TB treatment was a major challenge that leads to poor treatment outcomes. In Ethiopia, TB treatment success was low as compared with the global threshold. Despite various studies done in TB treatment adherence, little was known specifically in continuation phase where TB treatment is mainly patient-centered. This study aimed to determine adherence to TB treatment and its determinants among adult patients during continuation phase.

**Methods:**

We deployed a facility-based cross-sectional study design supplemented with qualitative data to explore perspectives of focal healthcare providers. The study population was all adult (≥18 years) TB patients enrolled in the continuation phase and focal healthcare workers in TB clinics. The study included 307 TB patients from 22 health facilities and nine TB focal healthcare providers purposively selected as key-informant. A short (11 questions) version Adherence to Refill and Medication Scale (ARMS) was used for measuring adherence. Data was collected using an interviewer-administered questionnaire and in-depth interview for qualitative data. Binary logistic regression was applied to identify factors associated with patient adherence. We followed a thematic analysis for the qualitative data. The audio data was transcribed, coded and categorized into themes using OpenCode software.

**Results:**

Among 307 participants, 64.2% (95% CI (58.6–69.4%) were adherent to TB treatment during continuation phase. A multi-variable analysis shown that secondary education (AOR = 4.138, 95% CI; 1.594–10.74); good provider-patient relationship (AOR = 1.863, 95% CI; 1.014–3.423); good knowledge on TB treatment (AOR = 1.845, 95% CI; 1.012–3.362) and middle family wealth (AOR = 2.646, 95% CI; 1.360–5.148) were significantly associated with adherence to TB treatment. The majority (58%) of patients mentioned forgetfulness, and followed by 17.3% of them traveling away from home without pills as major reasons for non-adherence to TB treatment.

**Conclusions:**

The study indicated that patients’ adherence to TB treatment remains low during continuation phase. The patient’s education level, knowledge, family wealth, and provider-patient relationship were found positively associated with patient adherence. Forgetfulness, traveling away, and feeling sick were major reasons for non-adherence to TB treatment. Interventional studies are needed on those factors to improve patient adherence to TB treatment during continuation phase.

## Background

TB treatment follows a Directly Observed Treatment, Short-course (DOTS) strategy with a combined course of antibiotics that include rifampicin (R), isoniazid (H), pyrazinamide (Z) and ethambutol (E) for 2 months of intensive phase followed by 4H + 4R for 4 months continuation phase [1–3]. During intensive phase, daily medication is expected to be delivered under healthcare provider’s observation. While, during continuation phase daily medication is supposed to be supported by community including family, relatives, neighbors and community health workers [4–6].

Stop TB Strategy focuses on universal access to patient-centered treatment and high-quality care and treatment support as the cornerstone of DOTS to improve treatment success rate [3]. Adherence to TB treatment is a regular and complete medication intake that gives individual TB patients the best chance of cure and also protects the community from the spread of TB [3]. Patients’ failure to adhere to TB treatment has been a global problem that results in poor treatment outcomes like, drug resistance, relapse, death, and increased health care costs [4, 7, 8]. In Ethiopia, recent evidences indicated the national pooled TB treatment success rate was low as compared with the global threshold [9–11]. The low treatment success rate was prevailing in Northern part of Ethiopia [11].

Non-adherence to TB treatment has been reported as a major challenge in Ethiopia [12–17]. Forgetfulness [12, 16, 18, 19]; poor provider-patient relationship as well as communication [19–21], poor knowledge towards TB treatment, distance to the health facility, adverse clinical experiences and alcohol intake [14, 18, 19, 22–25] were most commonly reported reasons for non-adherence to TB treatment. All the previous studies, however, pooled level of adherence from both intensive and continuation phase, where the treatment approach for intensive and continuation phases were quite different. In addition, most studies tried to assess TB treatment adherence from the patient’s perspectives, while healthcare providers’ perspectives have gotten little attention. Pill count has been predominantly used for measuring adherence by many studies, despite, its proven limitation of overestimating the level of patient adherence to TB treatment [26].

Some studies tried to analyze the treatment phase as a predictor variable for patient adherence to TB treatment and found that continuation phase was a risk factor for non-adherence [18, 19, 27]. That may indicated that non-adherence could be more common during the continuation phase of treatment. A recent finding in the study area also showed that adherence to TB treatment was 84% (183/218) in intensive and 66% (58/88) during continuation phases [19].

The strategies for the TB treatment adherence support were quite different during intensive and continuation phases. During the intensive phase, patients attend the nearby clinic every morning to swallow their pills under direct observation of healthcare providers that could motivate and enforce patients to adhere in their medication. Whereas, a continuation TB treatment phase is a self-managed (patient-centered) approach in which TB pills are daily taken by the patient at home with a weekly pill refilling schedule from the nearby health facility. So that during continuation phase patients could be reluctant to take pills at home and community-based treatment supporters may be busy in own competing business. The national TB treatment guideline suggests assigning treatment supporters including, family, health extension workers for each patient to support the treatment follow-up [27]. However, little is known about the challenges of community-based treatment support systems during the continuation phase. Above all, evidence was limited on factors affecting adherence to TB treatment during continuation phase.

Therefore, this study aimed to determine level of adherence and associated factors among adult TB patients enrolled in the continuation phase. It also aimed to explore TB focal person’s perceptions of patient adherence to TB treatment during the continuation treatment phase.

## Methods

### Study design

An Institutional based cross-sectional study was conducted with adult TB patients in the continuation treatment phase from March 9 to May 30, 2019. It was supplemented with qualitative data to explore the lived experiences and perspectives of the healthcare providers on patient adherence during the continuation phase.

### Study setting and participants

The study was conducted in 9 districts of Central Gondar Zone and Gondar town administration in Northwestern Ethiopia. Namely, Gondar Zuria, Tach Armachio, Wegera, East Dembia, West Belesa, Takusa, East Belesa, Alefa, and Gondar town administration were included in the study. The study area consists of 74 functional public health facilities (Health centers and Hospitals) serving approximately 2.9 million people in the area. Primary healthcare units that involves Health posts, Health Centers and district hospitals are mandated to provide TB treatment and care. In each health facility, a TB clinic has been established with at least one TB focal healthcare provider to regularly manage cases and follow their treatment. TB focal healthcare provider is a healthcare provider that received additional short-term training on TB and permanently assigned to TB Clinic.

The study population included all adult (≥18 years) TB patients enrolled in the continuation treatment phase as eligible participants. Patients with Multi-drug-resistant Tuberculosis (MDR-TB) and Extensively Drug-resistant Tuberculosis (XDR-TB) were not included in the study due to the distinct treatment period and approaches. All healthcare providers who were assigned as TB focal person in TB clinics were eligible for the key-informant interview.

### Sample size and sampling techniques

We calculated the sample size using a single population proportion formula with assumptions, including, proportion of patients adherent to TB medications (*P* = 66%) during the continuation phase [19]; margin of error (d = 5%), and 1.5 design effect. Considering the finite population and 10% non-response rate, the final sample size was 331 TB patients selected from 22 health facilities in the 9 districts. Facilities were randomly selected stratifying by urban (town administrations) and rural settings. All eligible TB patients were included during the study period. Nine TB focal healthcare providers were purposively selected for key-informant interview from nine different health facilities.

### Data collection tools and procedures

1. Description of interviewer-administered questionnaire and key-informant interviews

In this study, socio-demographic characteristics, disease characteristics, TB treatment-related knowledge, and attitude, provider-patient relationship using below and above the average score as a cut-off values. Family wealth quantiles were also included as predictor variables. We used an interviewer-administered questionnaire for socio-demographic, behavioral, and treatment adherence related data using Amharic (local language). But the treatment related information was taken from TB unit registers. The internal consistency of the tool was checked through piloting, hence, the Cronbach’s α value found to be 0.769, which was approaching well with the cut of value [28]. The content and face validity were also evaluated by six senior domain experts selected from TB treatment centers, TB experts in health offices, and behavioral researchers in research and teaching institutes. We trained and employed six data collectors and two supervisors. We assessed patients’ TB treatment-related knowledge using 8 questions that include, TB curability, how to confirm cure of the disease, TB treatment period, refilling time and adherence, feeding practice, side-effects, and treatment supporter’s role. Those patients who scored above the median value were determined as good knowledge [17, 19, 29, 30]. Similarly, a 4 attitude questions that include, patient trust on TB medication, medication related misconceptions, value for medication and belief of cure were used to assess attitude towards TB treatment [19, 20]. Those patients who score above the median value of attitude questions were determined as good attitude towards TB treatment. In order to measure provider-patient relationship, we used a 6 questions that include ways of communication, [19] were adapted from literatures. The family wealth quantiles (lowest, second, middle, fourth, highest) was constructed from multiple items including household assets, services, and facilities [31]. Then for simplicity of analysis, we merged into three outcomes (poor, middle and rich) by categorizing below middle quantiles into poor, middle quantiles as it is and above the middle quantiles into rich.

For the qualitative data, a semi-structured key informant interview guide was used to explore the existing provider-patient relationship and support during the continuation treatment phase. Participants were asked mainly about their opinion on which TB treatment phase is the risk for non-adherence? Why non-adherence to TB treatment was high on the specified phase? How was the communication and relationship between patients and healthcare providers? And how effective was the community-based treatment support system? The key-informant interview was recorder for audio after getting consent from each participants.

2) Description of assessment of the outcome

To assess adherence to TB treatment during continuation phase we deployed a short (11 questions) version of the Adherence to Refill and Medication Scale (ARMS) [32]. The original ARMS tool consists of 12 questions with two subscales, 8 questions about medication-taking, and 4 questions about refilling [33]. One of the items, “How often do you forget to take your medicine when you are supposed to take it more than once a day?” was not relevant to TB medication, since TB pills often are taken once a day. Each of the items were structured as a Likert scale with responses of “none,” “some,” “most,” or “all” of the time, which were given values from 1 to 4.

### Data analysis

We used a Principal Component Analysis (PCA) technique to compute the family wealth index quantile separately for urban and rural depending on assets and services specific to the urban and rural population [31].

Adherence to TB treatment was measured using the short (11 questions) version ARMS values that range from 1= “none of the time” to 4=“all of the time”. One item was reverse coded then the overall adherence score ranges from 11 to 44. The lower scores indicate better adherence and the higher score represents a higher level of non-adherence, items were asking about how frequently failed to adhere to specific elements [33]. The scales were further transformed into dichotomous outcomes using the recommended classification (scored 11 as adhered and > 11 as non-adhered) [32, 33].

We used the Variance Inflation Factor (VIF) to check the multicollinearity effect among predictor variables [34]. We conducted a single-level analysis using binary logistic regression to identify factors that are associated with adherence to TB treatment during the continuation phase. The Crude Odds Ratio (COR) and Adjusted Odds Ratio (AOR) with 95% CI and *p* value < 0.05 were computed using STATA version 14 software to determine statistical significance of the association between predictor and outcome variables.

For the qualitative data analysis, we transcribed the audio data into the Amharic language by experts in the field and translated into English by fluent speakers. After familiarization with the transcript, we assigned codes inductively and deductively and categorized into themes. We examine patterns, relationships, contradictory responses, and gaps in understanding in each theme. Quotes were selected and presented for norms of the participants’ shared perceptions.

## Results

### Socio-demographic and economic characteristics of participants

In this study, a total of 307 participants (92% response rate) were interviewed. More than half of the participants 178 (58.0%) were males. Of the total participants, 110 (35.8%) were aged between 35 and 44 years. The median age of the participants was 29.0 years with a minimum of 18 and a maximum of 84 years. Regarding marital status, 142 (46.3%) of the participants were single. The majority, 285 (92.8%) of them were Orthodox Christian followers. Of the total participants, 220 (71.7%) were urban residents. Among the participants, 101 (32.9%) could not read and write. The family asset evaluation shown that 62 (20.2%) were in lowest quantile. One hundred twenty two (39.7%) of the participants did not have a mobile phone at all (Table 1).

**Table 1:** Socio-demographic characteristics of participants, northwest Ethiopia (*n* = 307)

Characteristics	n (%)
Age	
Below 25	84 (27.4)
25–34	110 (35.8)
35–44	60 (19.5)
45+	53 (17.3)
Sex	
Male	178 (58.0)
Female	129 (42.0)
Marital status	
Single	142 (46.2)
Married	119 (38.8)
Divorced	34 (11.1)
Widowed	12 (3.9)
Religion	
Orthodox	285 (92.8)
Muslim	20 (6.5)
Others	2 (.7)
Residence	
Urban	220 (71.7)
Rural	87 (28.3)
Educational level	
Can’t read and write	101 (32.9)
Informally educated	37 (12.1)
Primary	73 (23.8)
Secondary	56 (18.2)
Higher	40 (13.0)
Partner’s educational level	
Can’t read and write	47 (39.5)
Informally educated	10 (8.4)
Primary	33 (27.7)
Secondary	17 (14.3)
Higher	12 (10.1)
Type of facility enrolled for treatment	
Health Center	267 (86.9)
Hospital	40 (13.1)
Family wealth quantile	
Lowest	62 (20.2)
Second	61 (19.9)
Middle	62 (20.2)
Fourth	61 (19.9)
Highest	61 (19.9)
Own a mobile phone	
Yes, smartphone	55 (17.9)
Yes, basic phone	130 (42.4)
Not at all	122 (39.7)

Of the nine key-informants, seven were selected from Health Centers and two from Hospitals. Six were males. The age of the participants ranged from 26 to 40 years. Only three of the participants had a first degree and above. Working experience ranged from 6 months to 7 years in TB clinic.

### Adherence to TB treatment

Overall, 197 (64.2%) of the participants were adherent (95% CI 58.6–69.4%) to TB treatment during continuation phase. The finding showed that 65 (34.8%) of pulmonary TB and 45 (37.5%) of extra pulmonary TB cases were adherent to TB treatment during continuation phase. One hundred three (35.9%) new and 7 (35.0%) repeated treatment cases were adherent to TB treatment (Table 2).

**Table 2:** Adherent to TB treatment by clinical characteristics of participants during continuation phase, northwest Ethiopia (*n* = 307)

Characteristics	Adherent to TB treatment	
	Yes	No
Type of TB		
Pulmonary TB	122 (65.2%)	65 (34.8%)
Extra pulmonary TB	75 (62.5%)	45 (37.5%)
Treatment category		
New	184 (64.1%)	103 (35.9%)
Relapse	13 (65.0%)	7 (35.0%)
Disclosed TB status to family		
Yes	189 (64.3%)	105 (35.7%)
No	8 (61.5%)	5 (38.5)
TB/HIV co-infected		
Yes	14 (53.9%)	12 (46.2%)
No	183 (65.1%)	98 (34.9%)
**Overall adherence**	**197 (64.2%)**	**110 (33.3)**

Focal TB healthcare providers were asked about “which treatment phase has a higher risk of non-adherence to the treatment?” Five of nine focal healthcare providers agreed that non-adherence to TB treatment worsens during the continuation phase as compared with the intensive phase, where patients take their daily pills at the clinic directly observed by the healthcare provider. One male TB focal healthcare provider replied that:

“*… when patients enrolled in the continuation phase, they often get reluctant to their pills. Let alone daily medication, they even miss weekly refilling appointments, however, during the intensive phase, they come to take their pills on time”*

### Determinants of adherence to TB treatment during continuation phase

The univariate logistic regression analysis showed that patient age, educational level, distance to the health facility, provider-patient relationship, patient knowledge on TB treatment, and family wealth index were associated with adherence to TB treatment during the continuation phase.

Whereas, in the multivariable binary logistic regression analysis, patients’ educational level, provider-patient relationship, patient knowledge on TB treatment, and family wealth index were associated with the outcome variable. Secondary level educated patients were about four times more likely to adhere to TB treatment as compared to non-educated patients (AOR = 4.138, 95% CI; 1.594–10.740). Similarly, patients who reported good provider-patient relationships were about two times more likely to adhere to the treatment than their counterparts, with (AOR = 1.863, 95% CI; 1.014–3.423). Those who have good knowledge of TB treatment were also more likely to adhere (AOR = 1.845, 95% CI; 1.012–3.362). Patients with middle family wealth were about three times more likely to adhere than patients with poor family wealth (AOR = 2.646, 95% CI; 1.360–5.148) (Table 3).

**Table 3:** Factors associated with adherence to TB medication and pill refilling during continuation phase in Northwest Ethiopia (*n* = 307)

Variables	Adherent		COR(95%CI)	AOR(95%CI)
	Yes	No		
Patient age				
24 years and below	58	26	2.317 (1.139–4.712)*	1.533 (.642–3.661)
25–34 years	81	29	2.901 (1.461–5.757)*	2.123 (.937–4.813)
35–44 years	32	28	1.187 (.566–2.487)	.879 (.378–2.043)
45+ years	26	27	1	1
Sex				
Male	113	65	1	1
Female	84	45	1.074 (.669–1.724)	1.251 (.707–2.214)
Residence				
Urban	146	74	1.393 (.836–2.320)	.764 (.374–1.559)
Rural	51	36	1	1
Educational level				
No education	53	48	1	1
Informal education	20	17	1.065 (.501–2.268)	.729 (.299–1.773)
Primary	45	28	1.456 (.789–2.685)	1.057 (.501–2.231)
Secondary	46	10	4.166 (1.895–9.157)*	4.138 (1.594–10.74)*
Higher	33	7	4.27 (1.728–10.55)*	2.795 (.970–8.052)
Distance to the health facility				
Less than 5 km	72	156	2.708 (1.206–6.081)*	2.275 (.877–5.903)
5-10 km	23	29	1.576 (.618–4.018)*	1.672 (.589–4.746)
Greater than 10 km	15	12	1	1
Treatment supporter assigned				
Yes	74	123	1	1
No	36	73	1.22 (.746–1.996)	1.375 (.769–2.457)
Disclosed TB status to family				
Yes	105	189	1.125 (.359–3.527)	.744 (.191–2.902)
No	5	8	1	1
Provider-patient relationship				
Good	112	45	1.903 (1.186–3.055)*	1.863 (1.014–3.423)*
Poor	85	65	1	1
Knowledge on TB treatment				
Good	157	72	2.072 (1.226–3.5)*	1.845 (1.012–3.362)*
Poor	40	38	1	1
Attitude on TB treatment				
Favorable	121	59	1.376 (.858–2.206)	1.272 (.702–2.305)
Unfavorable	76	51	1	
Family wealth index				
Poor	54	49	1	1
Middle	71	31	2.078 (1.173–3.683)*	2.646 (1.360–5.148)*
Rich	72	30	2.178 (1.225–3.871)*	1.949 (.957–3.968)

*p-value less than 0.05

**Fig. 1:**
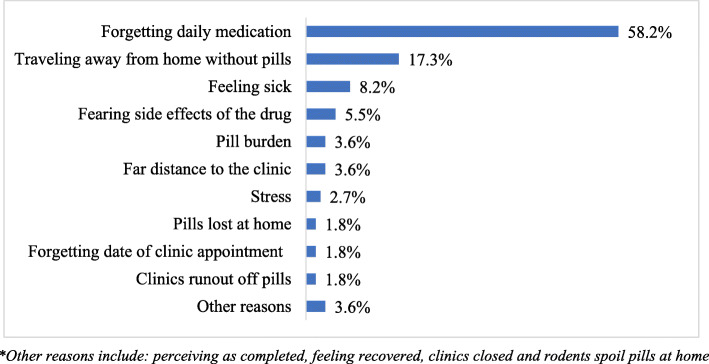
Patients’ reasons for non-adherence to TB treatment during continuation phase (*n* = 110). *Other reasons include: perceiving as completed, feeling recovered, clinics closed and rodents spoil pills at home

Patients who were non-adherent to their TB medication were asked for their reasons for failure to treatment adherence. More than half (58%) of 110 non-adherent participants reported forgetfulness to daily medication, 17.3% reported traveling away from home without pills, 8.2 and 5.5% were due to feeling sick and fearing side effects of the drugs, respectively (Fig. 1).

TB focal healthcare providers were also asked for their perspectives about contributing factors for non-adherence to TB treatment. Poor communication and relationship with their patients, transportation and related costs for pill refilling, relapsing of the disease, political unrest, and poor treatment support at the community were found major contributing factors for non-adherence to TB treatment during the continuation phase.

All respondents agreed that good communication and relationship was essential for patient adherence to TB medications. However, about half (4/9) of participants reported that their communication with patients declined as the patients shifted from the intensive phase (facility-based treatment) to the continuation phase. Another male TB focal healthcare provider also added that:

“During the continuation phase, we do not have daily contact with patients as we do on intensive phase. Even, weekly attendance was not easy. Some patients did not come, they send their supporter/family member to the clinic for refilling, and they were busy with family matters, social events like a funeral.”

In the key-informant interview, the majority of TB focal providers also exemplified that patients with low income tend to miss and/or interrupt refilling due to transportation and related costs. Besides, relapse cases tend to default treatments. A female TB focal healthcare provider replied that

“TB patients on continuation phase often miss refilling and from my experience, their reasons are mainly related to transportation cost and unintended social events”*.*

Participants also mentioned that relapsing cases tend to lost-to-follow-up. Another female focal healthcare provider added that:

“This year alone we lost two patients due to relapse of the case [TB]. One female insisted not to take the pills anymore and decided to go to Monastery. Similarly, one male patient lost from treatment follow-up …”

Political unrest and security problems were among the challenges for the patient, the health facility, and central drug suppliers (hubs for supplying drugs based on the health facilities’ need). A male TB focal healthcare provider replied that

*“Our community was victimized with frequent political turmoil and security problems which results in lost-to-follow due to massive displacement, migration of healthcare providers, and interruption of drug supply from the center.*”

Respondents were also asked their opinion on whether the assigned treatment supporters were helpful during the continuation treatment phase. Seven of nine participants replied that they were not helpful as intended, and (2/9) reported as helpful. The problem begins with assigning treatment supporters. A male TB focal healthcare provider reported that:

“During assigning treatment supporters, patients often choose educated relatives without considering the distance away from their home: mostly, they choose their relatives in urban while the patient living in rural.”

In addition, the commitment of treatment supporters was confronted by their income level and their prior commitments as reported by the respondents. A male TB focal healthcare provider mentioned that:

“Treatment supporters very rarely accompany patients during refilling, they mention transportation cost, own family, and social commitments.”

Another male TB focal healthcare provider also added that:

“During the continuation phase, we assign treatment supporters but in practice, most did not follow and most focal providers lack skills to influence patients to take their pills at home”

## Discussion

This study identified that adherence to TB treatment was low among adult TB patients during continuation phase in northwest Ethiopia. Patients’ educational level, knowledge, family wealth, and provider-patient relationship were positively associated with adherence to TB treatment. From the healthcare providers’ perspectives, poor communication and relationship with patients, transportation and related costs for refilling, relapsing of the disease, political unrest, and poor treatment support at the community were major underlying factors for non-adherence to TB treatment during the continuation phase.

The finding was similar with a study done in Gondar town of northwest Ethiopia [19]. Whereas, the finding was lower as compared to the pooled estimate of the national level of adherence to TB treatment [16]. Similarly, It was also lower as compared with other studies conducted in different parts of Ethiopia including in Arba Minch town [14], in Sidama [35], in Addis Ababa [15], in Alamata [12]. The discrepancy could be due to the current study included patients only from the continuation phase and other studies involved both intensive and continuation phases. And evidences indicated that non-adherence to TB treatment worsens during the continuation phase [18, 19, 27]. In this study, TB focal healthcare providers also have shown their agreement that the continuation phase was a risk for patients’ failure to adhere to TB treatment. Besides, we used a composite items to measure adherence to TB treatment. While, previous studies used pill count as a measure of adherence that often distorted the problem [26].

This study has shown that educational level was positively associated with adherence to TB treatment. The finding was in line with a study conducted in Equatorial Guinea [36] and Nepal [37] indicated that patient literacy was a significant factor for non-adherence to TB treatment. The current study has shown that patients with the middle family wealth index were about 3 times more likely to adhere to their TB medication as compared to patients in poor family wealth index. Patients with rich family wealth have also shown higher adherence as compared to patients in the poor category, however, it was marginally insignificant. The finding was in line with a study in Nepal [37]. Our qualitative finding also supported that transportation and related expenses during traveling for pill refilling were among the challenges that directly influence their adherence to the treatment.

Similarly, a good provider-patient relationship was also significantly associated with patient adherence during the continuation phase. The finding was consistent with previous studies which reported that provider-patient relationship was associated with adherence to TB treatment in Ethiopia [19–21]. Our qualitative analysis also revealed that healthcare providers did not have a strong relationship with their patients. Provider-patient relationship is considered as the core element of ethical principles of medicine that establish trust and motivation to engage patients on their treatment. The relationship could be a driver of good clinical outcomes. It promotes desired treatment results and also prevents adverse treatment outcomes [38, 39].

Patients with good knowledge related to TB treatment were about 2 times more likely to adhere to their medication and refilling. Similarly, studies in Ethiopia [17, 19] and China [30] also reported that poor knowledge about TB and TB treatment was significantly associated with non-adherence.

Generally, this study has shown that non-adherence to TB treatment was a problem during the continuation phase in the area. It implies that the level of adherence is different across treatment phases that could require to establish tailored strategies specific to continuation phases to improve patient adherence to TB treatment.

### Limitation of the study

This study specifically focused on continuation treatment phase that could give insight for policy, program and delivery of TB treatment and care. The limitation of the study includes use of self-reported adherence that often distort the problem due to recall and social desirability biases. Our study participants were enrolled from public health centres and hospitals. We did not address patients at private health facilities and health post levels. This could affect the generalizability of the study findings to the wider context. The ARMS tool looks to have very strict criteria in classifying adherence level. As a result, the level of adherence could be underestimated. We did not address all possible predictors of adherence to TB treatment during continuation phase. ARMS tool do not allow to classify adherence at data collection stage, it classifies after data analysis. Therefore, we asked about patient reasons for non-adherence to TB medication based on those missed one or more doses.

## Conclusion

TB treatment adherence was low during the continuation phase in northwest Ethiopia. Patients’ educational level, family wealth, provider-patient relationship, and patient knowledge related to TB treatment were positively associated with the level of adherence. Traditional and innovative interventional strategies would be useful to facilitate provider-patient relationships and TB treatment related knowledge of patients to improve their adherence to TB treatment during the continuation phase.

## Data Availability

The datasets used and/or analysed during the current study are available from the corresponding author on reasonable request.

## Abbreviations

ARMS: Adherence to Refill and Medication Scale
DOTS: Directly Observed Treatment, Short-course
HIV: Human Immunodeficiency Virus
MDR-TB: Multi-drug-resistant Tuberculosis
TB: Tuberculosis
XDR-TB: Extensively Drug-resistant Tuberculosis
